# Fate of Per- and Polyfluoroalkyl Substances from Durable Water-Repellent Clothing during Use

**DOI:** 10.1021/acs.est.1c07876

**Published:** 2022-04-11

**Authors:** Ike van der Veen, Steffen Schellenberger, Anne-Charlotte Hanning, Ann Stare, Jacob de Boer, Jana M. Weiss, Pim E. G. Leonards

**Affiliations:** † Department Environment and Health (E&H), Vrije Universiteit, De Boelelaan 1085, 1081 HV Amsterdam, The Netherlands; ‡ Department Environmental Science (ACES), Stockholm University, Svante Arrhenius väg 8, SE-11418 Stockholm, Sweden; § RISE, Research Institutes of Sweden, Brinellvägen 68, 100 44 Stockholm, Sweden; ∥ RISE IVF AB, Argongatan 30, SE-431 53 Mölndal, Sweden

**Keywords:** Side-chain fluorinated polymers, aging, washing, tumble drying, polyamine, polyester

## Abstract

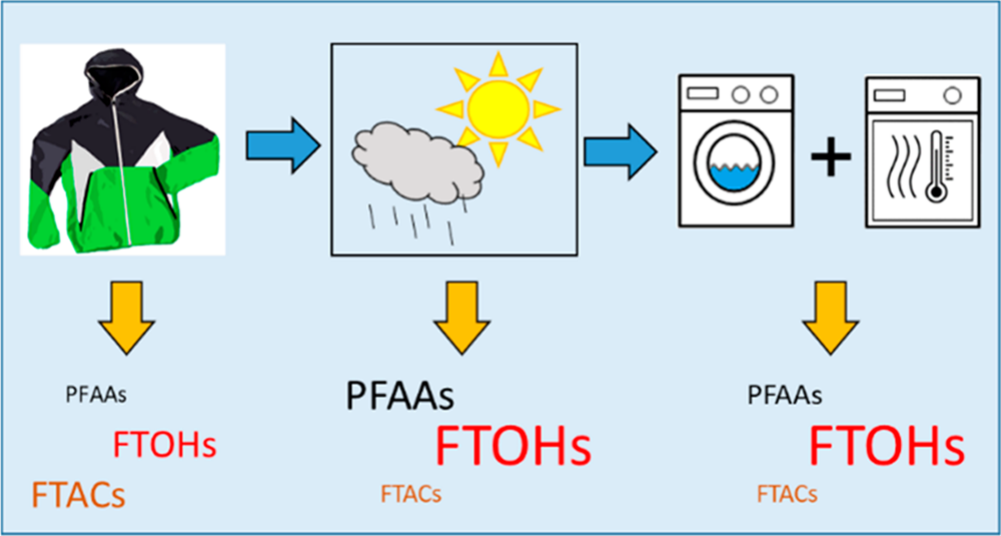

To make outdoor clothing water- or dirt-repellent, durable water-repellent (DWR) coatings based on side-chain fluorinated polymers (SFPs) are used. During use of outdoor clothing, per- and polyfluoroalkyl substances (PFASs) can be emitted from the DWR to the environment. In this study, the effects of aging, washing, and tumble drying on the concentration of extractable PFASs in the DWR of perfluorohexane-based short-chain SFPs (FC-6 chemistry) and of perfluorooctane-based long-chain SFPs (FC-8 chemistry) were assessed. For this purpose, polyamide (PA) and polyester (PES) fabrics were coated with FC-6- and FC-8-based DWRs. Results show that aging of the coated fabrics causes an increase in concentration and formation of perfluoroalkyl acids (PFAAs). The effect of aging on the volatile PFASs depends on the type of fabric. Washing causes a decrease in PFAA concentrations, and in general, volatile PFASs are partly washed out of the textiles. However, washing can also increase the extractable concentration of volatile PFASs in the fabrics. This effect becomes stronger by a combination of aging and washing. Tumble drying does not affect the PFAS concentrations in textiles. In conclusion, aging and washing of fabrics coated with the DWR based on SFPs release PFASs to the environment.

## Abstract 2

Aging and washing of outdoor clothing coated with durable water-repellent (DWR) coatings based on side-chain fluorinated polymers release very persistent per- and polyfluoroalkyl substances (PFASs) to the environment.

## Introduction

In outdoor clothes and workwear for protection (e.g., for fire fighter, emergency medical services), side-chain fluorinated polymers (SFPs) are being used because of their water- and oil-resistant properties. The SFP structure consists of a backbone of polymers such as polyurethanes or acrylates. To this backbone, per- and polyfluoroalkyl substances (PFASs) are bound as side chains, which are usually based on fluorotelomer alcohols (FTOHs), fluorotelomer acrylates (FTACs), and fluorotelomer methacrylates (FTMACs).^1,2^ By abiotic and biotic degradation, FTOHs, FTACs, and FTMACs can degrade and (bio)transform into perfluoroalkyl acids (PFAAs), which are very persistent and very mobile in the environment.^3−12^ Some PFASs, such as perfluorooctanoic acid (PFOA), have been shown to cause adverse health effects, like liver damage, increased cholesterol levels, and a lower immune response after vaccination.^13,14^ Some individual PFASs have been regulated in Europe. Perfluorooctanesulfonate (PFOS) and PFOA and related substances are restricted under the EU POPs Regulation and listed under the Stockholm Convention on persistent organic pollutants (POPs).^15−17^ Perfluorohexanesulfonate (PFHxS) and some long-chain perfluoroalkyl carboxylic acids (PFCAs) (C9–C14) are proposed for listing as POPs under the Stockholm Convention.^18−20^ The maximum limit for PFOA in products, including textiles, is 0.025 mg/kg, with an exception for textiles used in protective workwear until July 4, 2023.^21^ Because of the high persistence of PFASs, industries started to phase out the use of some of the longer-chain PFCAs and perfluoroalkanesulfonic acids (PFSAs).^1,22,23^ This led to the production and use of alternative compounds to obtain the required durable water repellency (DWR) for outdoor clothing and workwear. Some of the alternatives now brought to the market were silicones and waxes but also shorter-chain PFASs.^1,2,24^ To assess the emissions of DWR components, e.g., old-fashioned but phased out long-chain PFASs, and alternative chemistries to the environment and to assess the functionality of the alternatives compared to long-chain PFASs, the SUPFES (Substitution in Practice of Prioritized Fluorinated Chemicals to Eliminate Diffuse Sources) project was initiated in 2013.^25^ After assessing the performance of different types of DWR, it was concluded by Schellenberger et al.^2^ that the alternative chemistries, like silicones or waxes, could deliver the desired water repellence. However, it was not possible to achieve the oil- and dirt-repellent properties that PFASs can deliver. Although the DWR coating of fabrics consists mainly of SFPs^1,26^ after coating, some unreacted ionic or volatile PFAS residuals or impurities might still be present on the fabrics.^27^ Several studies are published on PFAS concentrations in textiles,^27−38^ and a variety of studies are published on the emission of PFASs to the environment.^39−46^ Recently, it has been reported that aging of textiles is one of the factors that influences the fate of PFASs in outdoor clothing during use.^27^ Aging of textiles with a DWR-based PFAS chemistry can lead to an increase in some of the extractable PFAS concentrations. DWRs can contain known and unknown impurities from production which are precursors of PFAAs. A possible explanation of the increase in concentrations of extractable PFASs by aging might be that some of the unknown impurities are transformed or degraded by aging into some of the target PFASs. Other possibilities for emission is side-chain cleavage of the SFPs or the release of nonextractable organic fluorine (NEOF). Other factors influencing the fate of PFASs during the use of outdoor clothing are washing and tumble drying of the clothing. Although the effect of aging on PFASs in the DWR of textiles of outdoor clothing has been described previously,^27^ to the best of our knowledge, the effects of washing and tumble drying in combination with aging on PFCAs, FTOHs, FTACs, and FTMACs in the DWR coated textiles have not been assessed before. However, the effect of one washing cycle and tumble drying on FTOHs in two treated home textiles and upholstery products was assessed by Liu et al.^47^ Knepper et al.^48^ reported in a nonpeer reviewed report PFAA concentrations in washing water after washing four pieces of outdoor jackets together. Commercially available textiles of outdoor clothing are less suitable to make a good comparison between different DWR chemistries, because it is often unknown what type of DWR chemistry was applied on those textiles and which other additives are present. Therefore, in the SUPFES project,^25^ two fabrics, a polyamide (PA) textile and a polyester (PES) textile, have been coated with different PFAS-based DWR formulations provided by major raw material suppliers of DWR chemicals and by following processes close to conditions used by textile manufacturers.^2^ In our study, the textiles were subjected to accelerated weathering under laboratory conditions, simulating the outdoor exposure of textiles to weather conditions, and a number of washing plus tumble drying cycles. Within the SUPFES project,^25^ the functionality of those textiles before and after aging, washing, and tumble drying was assessed.^2^ The aim of the present study was to assess the effect of washing, tumble drying, and aging on the PFASs in the DWR of perfluorohexane-based short-chain SFP (FC-6 chemistry) coated textiles compared to the effect on the PFASs in the old-fashioned but phased out perfluorooctane-based long-chain SFP (FC-8 chemistry) coated textiles. A comparison was made between the concentration and identity of PFASs before and after aging, washing, and tumble drying cycles. A perfluorobutane-based SFP (FC-4 chemistry) coated PES fabric was evaluated for homogeneity to demonstrate the quality of the coating method applied in the project. The studied PFASs are the ionic PFAAs including the C_4_–C_14_ PFCAs and the C_4_, C_6_, C_7_, and C_8_ PFSAs. The volatile PFASs studied are the *n*:2 FTOHs (4:2, 6:2, 8:2, 10:2), the *n*:2 FTACs (6:2, 8:2, 10:2), and the *n*:2 FTMACs (6,2, 8:2, 10:2).

## Materials and Methods

The effect of aging, washing, and tumble drying on PFASs in the DWR coated fabrics was assessed on four DWR coated fabrics. Aging of textiles can only be performed on small pieces of textiles, which are needed in total for PFAS analysis to meet the limit of detection (LOD). In addition, the coated fabrics were needed for additional performance testing as well.^2^ This resulted in a limited available amount of treated fabrics. Because the washing and tumble drying were carried out according to ISO protocols,^49,50^ and because we performed the weathering before in an earlier study^27^ and found the same patterns, we considered duplicate analyses redundant. In addition, to secure the same results, the washing machine was calibrated and checked concerning quantity of water and temperature, before performing the washing of the fabrics.

### Chemicals and Reagents

The PFAAs and volatile PFASs assessed and analyzed in this study are given in Tables S1 (PFAAs) and S2 (volatile PFASs) of the Supporting Information (SI). PFAAs (50 μg/mL in methanol) were obtained from Greyhound Chromatography (Merseyside, UK). Volatile PFASs (50 μg/mL in methanol) were purchased from Chiron AS (Trondheim, Norway). Ultrapure water originated from a Milli-Q system from Millipore (Watford, UK). Ethyl acetate (HPLC, 054006) was supplied by Biosolve Chimie (Dieuze, France). Acetonitrile (Chromasolve, 34851), Supelclean Envi-carb (Supelco, 957210-U), and ammonium formate (Bio ultra, 09735) were purchased from Sigma-Aldrich (Zwijndrecht, The Netherlands). HPLC grade acetone (J.T. Baker, 9254) and methanol (J.T. Baker, 8402) were obtained from Boom (Meppel, The Netherlands).

### Fabrics

Two types of synthetic fabrics, a PA fabric and a PES fabric, which are regularly used for the production of outdoor clothing, have been provided by FOV AB, Borås, Sweden (SI Table S3). To both types of fabrics, DWR coatings based on FC-6 chemistry and FC-8 chemistry have been applied as described by Schellenberger et al.,^2^ and to the PES fabric based on FC-4 chemistry, the DWR coating has been applied (SI Table S3).

### DWR Textile Treatments

The effects of aging, washing, and tumble drying were assessed on the PA and PES fabrics coated with the FC-6 and FC-8 DWR emulsions. Since the FC-4 coated fabrics did not meet the criteria for performance testing on abrasion, oil repellence, and water repellence after aging,^2^ those materials were not considered to be a good alternative for the FC-8 coatings and hence were not included in this study. An overview of all treatments and the number of samples analyzed per treatment can be found in Table 1 and are described below.

**Table 1:** Treatments of PA and PES Textiles Coated with FC-6 and FC-8 Chemistries Expressed in Numbers of Samples Analyzed

			no. of samples					
sample code	DWR chemistry	fabric	PFAAs	volatile PFASs		aged	washing^a^	tumble drying^a^
1-7	FC-6	PA	5	2		no	no	no
8-9	FC-6	PA	1	1		yes	no	no
10-11	FC-6	PA	1	1		yes	5 cycles	5 cycles
12-13	FC-6	PA	1	1		yes	10 cycles	10 cycles
14-15	FC-6	PA	1	1		yes	5 cycles	no
16-17	FC-6	PA	1	1		no	5 cycles	5 cycles
18-19	FC-6	PA	1	1		no	no	5 cycles
								
20-26	FC-6	PES	5	2		no	no	no
27-28	FC-6	PES	1	1		yes	no	no
29-30	FC-6	PES	1	1		yes	5 cycles	5 cycles
31-32	FC-6	PES	1	1		yes	10 cycles	10 cycles
								
33-39	FC-8	PA	5	2		no	no	no
40-41	FC-8	PA	1	1		yes	no	no
42-43	FC-8	PA	1	1		yes	5 cycles	5 cycles
44-45	FC-8	PA	1	1		yes	10 cycles	10 cycles
								
46-52	FC-8	PES	5	2		no	no	no
53-54	FC-8	PES	1	1		yes	no	no
55-56	FC-8	PES	1	1		yes	5 cycles	5 cycles
57-58	FC-8	PES	1	1		yes	10 cycles	10 cycles

a In case both washing and tumble drying were performed, one cycle consisted of washing followed by tumble drying.

#### Aging

The fabrics (FC-6 and FC-8 coated PA and PES fabrics) were aged in an ATLAS weather-o-meter Cr 3000 using the method previously described in Van der Veen et al.^27^ (SI Table S4). The fabrics were exposed to elevated temperatures, humidity, and UV irradiation for 300 h, which simulates exposure to weather conditions during a lifetime wear of outdoor clothing.

#### Washing and Tumble drying

The effect of washing plus tumble drying on the aged FC-6 and FC-8 coated fabrics was assessed after five and 10 sequential washing plus tumble drying cycles. Washing and tumble drying of the fabrics were performed according to SS-EN ISO 6330:2012^49^ and as described by Schellenberger et al.^2^ Each type of coated fabric was washed separately at 40 °C. Tumble drying was performed at 60 °C for 30 min.

Three additional assessments have been performed on the FC-6 coated PA fabric (Table 1). For the first assessment, five washing cycles without tumble drying were performed on the aged material. The second assessment contained five sequential washing plus tumble drying cycles on the original, not the weathered coated fabric. In the third assessment, five sequential tumble drying cycles were performed on the original, not the weathered coated material, without washing the fabric.

### Extraction and Instrumental Analyses

After each treatment (Table 1), the fabrics were analyzed for PFAAs and volatile PFASs. PFAAs were extracted and analyzed by the method earlier developed and described by Van der Veen et al.^38^ In short, textile samples of approximately 20 cm^2^, cut in smaller pieces, were extracted with methanol for the determination of PFAAs with an Agilent 6410 Triple Quad liquid chromatography-tandem mass spectrometer (LC-MS/MS, Agilent Technologies, Amstelveen, The Netherlands) in the electrospray negative ionization mode. For extraction and analysis of the volatile PFASs, the method described by Van der Veen et al.^27^ was used. In short, textile samples of approximately 20 cm^2^ were extracted with ethyl acetate. After cleaning the extracts with Envi-carb and a concentrating step, the extracts were analyzed with gas chromatography/electron impact-mass spectrometry (GC/EI-MS) on an Agilent 6890 series GC coupled to a 5973 Network MS (Agilent Technologies, Amstelveen, The Netherlands).

### Quality Control

#### Homogeneity of PFAS in the DWR Coated Fabrics

The homogeneity testing of the FC-4 coated PES fabric and of the FC-6 and FC-8 coated PA and PES fabrics is described in Chapter 2 of the SI. Because of the limited amount of fabric available, it was not possible to perform an extensive homogeneity test for each of the coated fabrics of interest in our study. Since the FC-6 and FC-8 coatings were applied by the same procedures as the FC-4 coating, it is likely that they were evenly coated as well. Therefore, a general assessment of the distribution of PFAS concentrations in the coated fabrics, and between the coated fabrics, was performed on PFAA concentrations in the PES fabrics coated with the FC-4 emulsion. For this, 20 samples were analyzed out of one piece of the FC-4 coated PES fabric (40 × 35 cm), and 10 samples were analyzed out of another piece (40 × 35 cm) of the FC-4 coated PES fabric (SI Figure S1). For the remaining four fabrics (FC-6 and FC-8 coated PA and PES fabrics), five samples (approximately 20 cm^2^) of each of the fabrics were analyzed for PFAAs, and two samples were analyzed for volatile PFAS concentrations.

#### Carryover in the Aging Device

In a previous study by Van der Veen et al.,^27^ the possible carryover of PFASs between the DWR coated fabrics in the aging device was determined.

## Results and Discussion

### Homogeneity of PFAS in the DWR Coated Fabrics

Results, as determined with the SoftCRM software,^51^ showed a homogeneous distribution of perfluorobutanoic acid (PFBA) over both fabrics of the FC-4 coated PES fabrics at the 99% confidence level (SI Table S5). The relative standard deviation (RSD) over 30 measurements was 14%. These results showed that coating fabrics with DWR emulsions based on SFPs by the method of Schellenberger et al.^2^ results in fabrics with a homogeneous PFAS distribution. This makes the fabrics suitable for the determination of the effect of aging, washing, and tumble drying on PFASs in the fabrics. The mean RSDs of all PFASs in the four coated fabrics of interest in our study (FC-6 and FC-8 coated PA and PES fabrics) were 25% (SI Figures S2–S6). This included the high RSDs of perfluorohexanoic acid (PFHxA) (95%) for the FC-6 coated PES fabric and of PFOA (63%) and perfluorononanoic acid (PFNA) (57%) for the FC-8 coated PA fabric. The RSDs of these limited homogeneity tests were taken into account in the evaluation of the results obtained from the aging, washing, and tumble drying studies.

### Initial PFAS Concentrations in the DWR Coated Fabrics before Treatments

Detailed information on the PFAS concentrations in the four DWR coated fabrics before and after aging, washing, and tumble drying experiments is shown in Table S6 of the Supporting Information.

#### PA versus PES Fabrics

**Figure 1:**
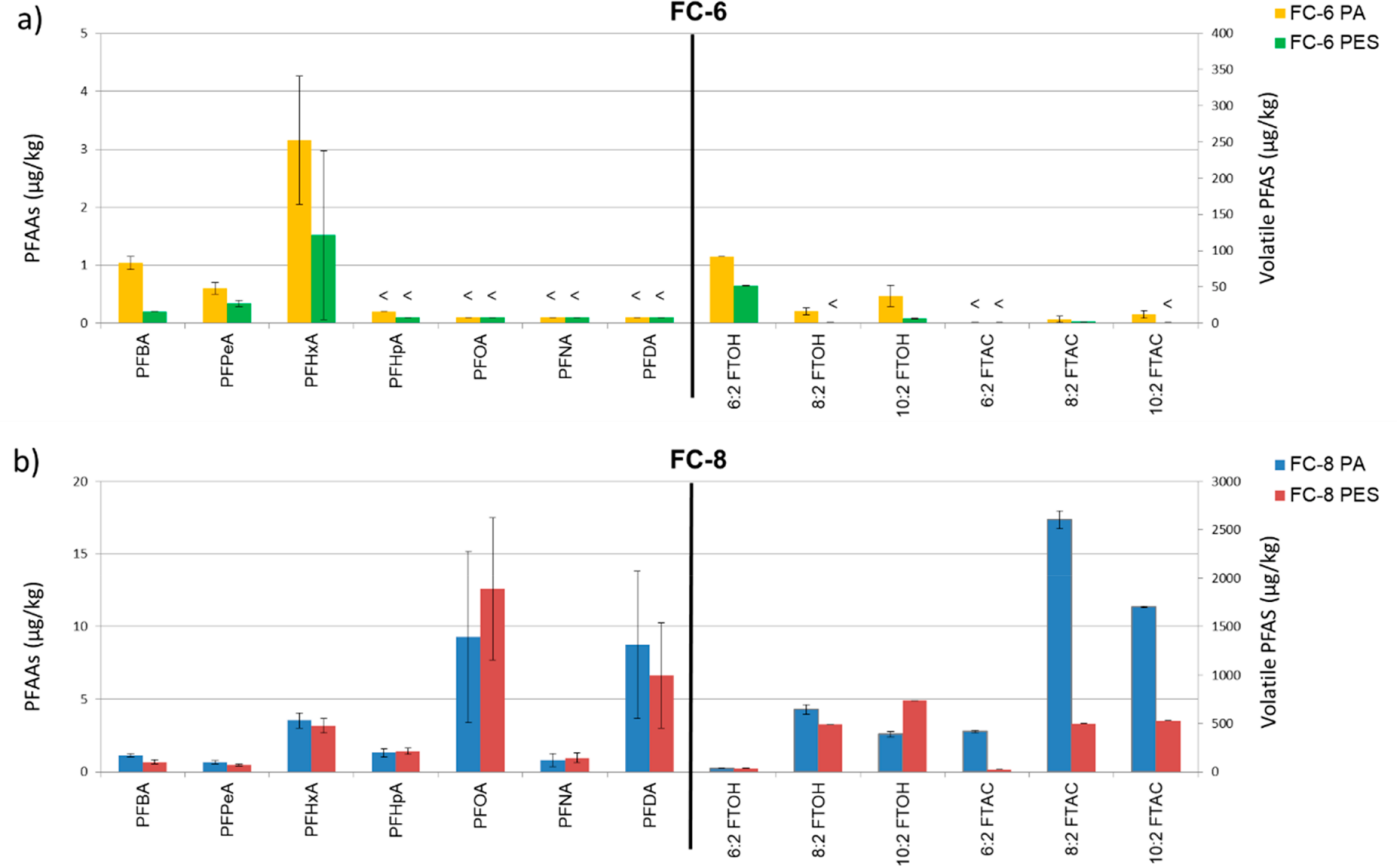
PFAS concentrations (μg/kg) of relevant PFASs in a PA and a PES fabric applied with a) a fluorocarbon 6 (FC-6) DWR emulsion and b) a fluorocarbon 8 (FC-8) DWR emulsion before aging, washing, and tumble drying. <: LOD.

In Figure 1, all PFAS concentrations detected in the coated PA and PES fabrics are given (seven PFAAs and six volatile PFASs). Those concentrations in the coated PA and PES fabrics were different even though the fabrics were coated with the same DWR emulsions. The FC-6 coated PA contained more volatile PFAS congeners than the FC-6 coated PES, and the concentrations of the PFASs which were present in both materials were two to six times higher in the FC-6 coated PA. In the FC-8 coated materials, the same PFAS congeners were detected in both the PA fabric and the PES fabric. Also, for this formulation, the concentrations of all detected volatile PFASs were higher in the PA fabric than in the PES fabric, except for 10:2 FTOH. In conclusion, the coated PA fabrics both contained more PFASsa then the PES fabrics. This difference in PFAS concentrations could be explained by the DWR uptake of the fabrics during the coating process. The PA fabric had a different weave structure (rib-stop pattern) than the PES fabric (plain weave) (SI Table S3). Another explanation might be the difference in hydrophobicity of PA compared to PES.^52^ Higher PFAS concentrations in PA fabrics compared to PES fabrics were also observed in the results of the studies of Gremmel et al.^34^ and of Santen et al.^29^ In both studies, commercially available outdoor jackets were analyzed for their PFAS content. In the study of Gremmel et al.,^34^ the sum of PFAS concentrations in PES textiles was 0.35–76.1 μg/kg (median 14.4 μg/kg), and the sum of PFAS concentrations in PA textiles was 62.8–500 μg/kg (median 145 μg/kg). In the study of Santen et al.,^29^ the sum of PFAS concentrations in PES textiles was 2.1–74 μg/m^2^ (median 23 μg/m^2^), and the sum of PFAS concentrations in PA textiles was 6.7–421 μg/m^2^ (median 37 μg/m^2^). In other studies on PFASs in the DWR coated fabrics, the types of fabrics were either not given,^28,30,31,36,37^ or no PA fabric was analyzed.^35^

#### FC-6 versus FC-8 DWR Coated Fabrics

The FC-8 coated fabrics contained more PFAAs congeners (PFBA, perfluoropentanoic acid (PFPeA), PFHxA, perfluoroheptanoic acid (PFHpA), PFOA, PFNA, and perfluorodecanoic acid (PFDA)) than the FC-6 coated fabrics, in which only three PFAAs congeners (PFBA, PFPeA, and PFHxA) could be quantified. The highest PFAA concentrations in the FC-6 coated fabrics were found for PFHxA (PA: 3.2 μg/kg; PES 1.5 μg/kg), and the highest volatile PFAS concentrations were found for 6:2 FTOH (PA: 92 μg/kg; PES 52 μg/kg). This result could be expected since the formulation used to coat the fabrics was based on FC-6 chemistry,^2^ and after coating, some unreacted ionic or volatile FC-6 PFASs residuals or impurities might still be present on the fabrics.^27^

In the FC-8 coated fabrics, besides the PFASs with a chain length of eight carbons (PFOA, 8:2 FTOH, and 8:2 FTAC), PFASs with a chain length of 10 carbons (PFDA, 10:2 FTOH, and 10:2 FTAC) were present in comparable concentrations. This might be due to the fact that DWR emulsions used for coating the fabrics often consist of a mixture of the desired SFPs and fluorinated polymers with shorter and longer side chains as a result of the production process.^3,26^ Other PFAAs (PFBA, PFPeA, PFHxA, PFHpA, PFNA) and volatile PFASs (6:2 FTOH, 6:2 FTAC) were detected in lower concentrations in the FC-8 coated fabrics. The FC-6 coated fabrics contained only two other PFAAs (PFBA and PFPeA). Four of the volatile PFASs (8:2 FTOH, 10:2 FTOH, 8:2 FTAC, and 10:2 FTAC) were detected on at least one of the FC-6 coated fabrics but all in much lower concentrations (2.4–38 μg/kg) than in the FC-8 coated fabrics (26–2600 μg/kg).

### The Effect of Aging, Washing, and Tumble Drying on PFASs in the DWR Coated Fabrics

**Figure 2:**
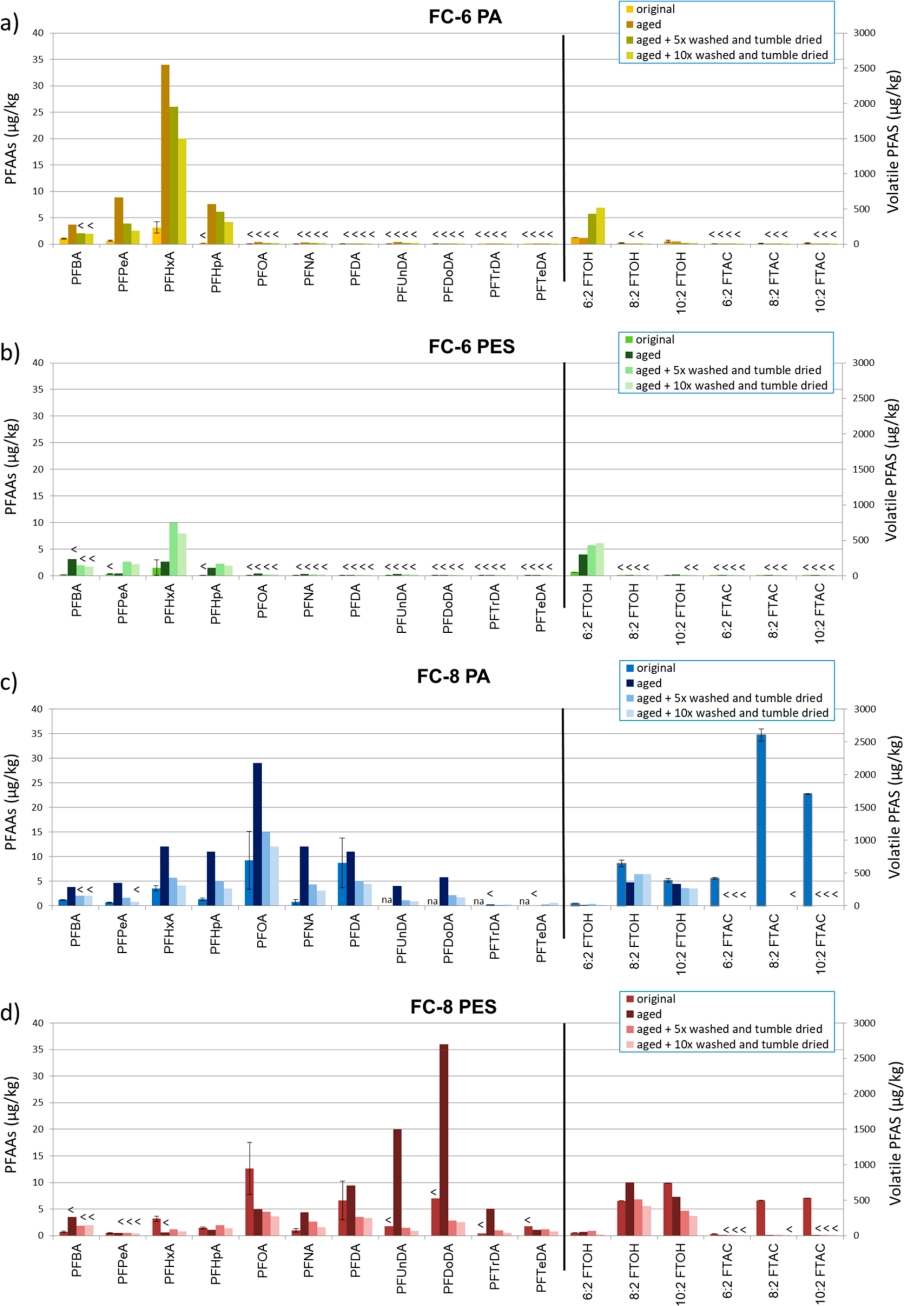
Effect of aging, washing, and tumble drying on PFAS concentrations (μg/kg) in a) a polyamide (PA) fabric applied with a FC-6 DWR emulsion, b) a polyester (PES) fabric applied with a FC-6 DWR emulsion, c) a PA fabric applied with a FC-8 DWR emulsion, and d) a PES fabric applied with a FC-8 DWR emulsion (na: not available due to low IS recovery). <: LOD.

In Figure 2, the PFAS concentrations in the FC-6 and the FC-8 coated PA and PES fabrics are shown before the textiles were aged (original), after aging, after aging followed by five times washing plus tumble drying cycles, and after the textiles were aged followed by ten washing plus tumble drying cycles.

#### The Effect of Aging on PFAAs

Aging of the FC-6 coated PA increased the concentrations of PFAAs which were present in the original coated fabric (PFBA, PFPeA and PFHxA) by a factor of 3.6–15. In addition, PFHpA was detected in the aged material (7.6 μg/kg), while this compound was not present in the original coated material (Figure 2). Also, in the aged FC-6 coated PES fabric, the concentration of PFHxA increased, and PFHpA was detected, although both were detected in lower concentrations (2.6 and 1.5 μg/kg, respectively) than in the PA fabric.

In the FC-8 coated PA fabric, an increase of 3–15 times in concentration of all detected PFAAs was observed after aging, of which especially the odd-chain PFASs were formed with an 6.8–15-fold increase, compared to the 1.3–3.4-fold increase of the even-chain PFASs. In the FC-8 coated PES fabric, the concentrations of longer-chain PFAAs (>C9) also increased. However, in this coated fabric, the concentrations of shorter-chain PFAS (C6–C8) decreased. In a previous study by Van der Veen et al.,^27^ the possible carryover of PFASs between the DWR coated fabrics in the aging device was determined. No carryover was observed for PFAAs.

The increase in PFAA concentrations and the formation of PFAAs by aging were in line with the findings of Van der Veen et al.^27^ on the aging of the commercially available DWR coated textiles of outdoor clothing. Also, the formation of odd-chain PFASs was observed in two samples in that study. The increase in extractable PFAA concentrations could be explained by atmospheric oxidation of FTOHs which were present in the coated fabrics,^9^ degradation, or transformation of other PFAA precursors, release of NEOF, or cleavage of the side chains of the SFPs.^27,53−55^

There is a difference between the effect of aging on the PFAAs in the FC-6 DWR chemistry and in the FC-8 DWR chemistry. The FC-6 coated materials only contained shorter-chain PFAAs with PFHxA being the PFAA with the longest carbon chain length (C_6_). After aging, the PFAA with the longest chain length was PFHpA (C_7_). In the FC-8 coated fabrics, the longest PFAA before aging was PFDA (C_10_). After aging, PFAAs with even longer carbon chain lengths appeared (PA: C_11_ and C_12_; PES: C_11_–C_14_).

To summarize, comparison of the PA and PES fabrics showed that aging of the coated PA fabrics resulted in an increase in concentrations of all PFAAs present in the original coated fabrics, and in addition, some PFAAs showed up which were not detected before aging. Aging of the coated PES fabrics resulted in a decrease of concentrations or even absence of shorter-chain PFAAs and an increase in concentration or appearance of PFAAs with a longer carbon chain (for PA: C6 and C7; for PES: C9–C14). The results show that shorter-chain PFAA residuals, impurities, or degradation products out of the DWR formulations more easily remained on the original coated and aged PA textiles than on the PES fabrics. The coated PES fabrics, on the other hand, gained more in concentration of longer-chain PFAAs. The difference in the weave structure of the PA fabric and the PES fabric (SI Table S3) might have influenced the coating process and explained this phenomenon. Also, the higher hydrophobicity of PES compared to PA^52^ might explain those results. The higher hydrophobicity results in a lower interaction to more hydrophilic short-chain PFAS. This could result in an easier release during weathering and lower their concentrations.

#### The Effect of Aging on Volatile PFASs

Aging resulted in the disappearance of all FTACs which were present in the coated fabrics before aging. This disappearance could be explained by atmospheric oxidation of the FTACs by reaction with OH radicals, which results in the formation of PFAAs as described by Butt et al.,^56^ or by hydrolysis with water, which forms FTOHs.^57^ Aging of the FC-6 coated fabrics, however, did not have an effect on the concentration of 6:2 FTOH in the PA fabric, but in the PES fabric, the concentration of 6:2 FTOH increased from 52 to 300 μg/kg. Also, in the FC-8 coated PES fabric, the concentration of the relevant FTOH (8:2 FTOH) increased from 490 μg/kg up to 750 μg/kg. A decrease of 60% was observed for the concentration of 8:2 FTOH in the PA fabric as an effect of aging. The possible carryover of volatile PFASs between the DWR coated fabrics in the aging device was determined in a previous study by Van der Veen et al.^27^ Of the investigated volatile PFASs, 6:2 FTOH (17 μg/kg), 8:2 FTOH (35 μg/kg), and 10:2 FTOH (35 μg/kg) were detected. Aging can have an effect on the DWR coatings, but it can also degrade PA and PES at the molecular level and change the properties of the textiles.^58,59^ Aging in our study could have released the unextractable fraction of 6:2 FTOH and 8:2 FTOH in the PES fabrics, while it did not in the PA fabrics. The effects of aging on 6:2 FTOH and 8:2 FTOH in our study are in agreement with the findings of Van der Veen et al.^27^ In that study, the concentration of 6:2 FTOH increased in 12 out of 13 textile samples after aging, and the concentration of 8:2 FTOH increased in some of the samples and decreased in other samples.

#### The Effect of Washing Plus Tumble Drying

Performing five washing plus tumble drying cycles on the aged fabrics resulted in a decrease in concentration of all extractable PFAAs in all coated fabrics; however, no conclusions can be drawn on the PFDA concentration due to the high variance in the original samples (Figure 2). Performing ten washing plus tumble drying cycles on the aged fabrics resulted in even lower concentrations of extractable PFAAs. The only exceptions to this decrease are PFHxA and PFHpA in the PES fabrics. However, due to the high RSDs detected over the analyses of five untreated coated fabrics, for PFHxA and PFHpA, no definitive conclusions can be drawn on the small increase in those two compounds after the first five washing plus tumble drying cycles.

Like the PFAAs, the 10:2 FTOH concentration decreased in all coated fabrics when five washing plus tumble drying cycles were performed after aging and decreased even further when ten washing plus tumble drying cycles were performed (Figure 2). Also, 8:2 FTOH in the FC-8 coated PES fabric followed this pattern. However, the concentration of 8:2 FTOH in the FC-8 coated PA fabric increased from 360 μg/kg to 480 μg/kg, and the concentration of 6:2 FTOH in both the FC-6 coated PA and PES fabrics increased from 87 to 430 μg/kg and from 300 to 430 μg/kg, respectively. This increase could be explained by the hydrolyses of residuals, impurities, or SFPs out of the DWR in combination with abrasion of the DWR coating or abrasion of the textile fibers, which occurs during the washing process.^57^ An explanation for the difference in observed effects for 8:2 FTOH in the PA and PES fabrics might be found in the type of fabric, since the DWR coating on both types of materials was the same. Washing and tumble drying of the aged FC-8 PES fabric most likely washes off 8:2 FTOH which was released by aging (see above), while washing and tumble drying of the aged FC-8 PA fabric did release more unextractable 8:2 FTOH than the amount of 8:2 FTOH which was washed off. The same phenomenon was observed by the 6:2 FTOH concentrations detected in the FC-6 fabrics. Washing plus tumble drying of the aged PA fabric did release unextractable 6:2 FTOH or did transform FTOH precursors. Similar to 8:2 FTOH in the FC-8 PES fabric, the concentration of 6:2 FTOH in the FC-6 PES fabric increased after washing and tumble drying of the aged fabric, however not so much as in the PA fabric.

This increase in concentration might be caused by a remainder of the unextractable fraction of 6:2 FTOH which became available by either washing or tumble drying. Another explanation of the further increase might be precursors which could transform into 6:2 FTOH as result of washing plus tumble drying as earlier described by Van der Veen et al.,^27^ as a possible explanation for the observed increase in 6:2 FTOH concentration as an effect of aging, or the cleavage of side chains of fluorotelomer-based polymer (FTPs). In the study of Liu et al.,^47^ no increase or significant losses of FTOHs were observed on the two assessed products. However, the washing and tumble drying in their study was limited to one washing and tumble drying cycle.

#### The Effect of Aging, Washing, and Tumble Drying on PFASs in the DWR Coated Fabrics Illustrated by 6:2 FTOH

**Figure 3:**
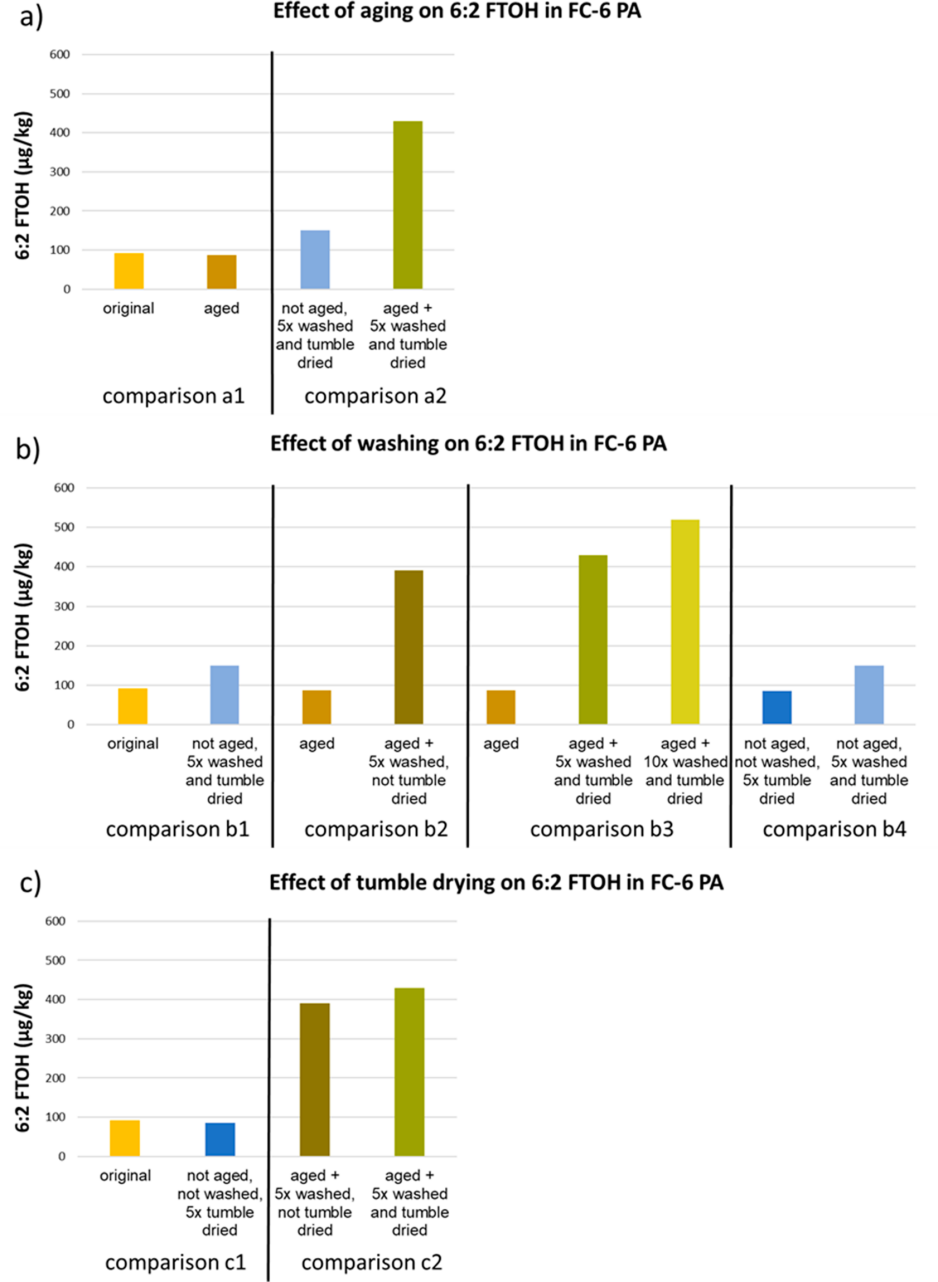
Effect of a) aging, b) washing, and c) tumble drying on the 6:2 FTOH concentration (μg/kg) in the PA fabric coated with a FC-6 DWR emulsion. To clearly show the effects of aging, washing, and tumble drying, different comparisons have been made between the analyzed samples. Corresponding colors represent the same analyses.

First, to assess whether the increase in 6:2 FTOH after aging, washing, and tumble drying was caused by either the effect of washing or the effect of tumble drying and second, to assess whether this increase also appears when not aged fabric would have been washed and tumble dried, some additional tests were performed on the FC-6 coated PA fabric. All PFAS concentrations detected in the original FC-6 coated PA fabric and in the aged, washed, and tumble dried FC-6 coated PA fabrics are shown in Figure S7 of the SI. In Figure 3a, the effects of aging on 6:2 FTOH in the FC-6 coated PA fabric are shown for two different treatments. When the original coated material was aged, no effect was observed in the concentration of 6:2 FTOH (Figure 3, comparison a1). However, washing plus tumble drying of the aged fabric results in a higher concentration of 6:2 FTOH than washing plus tumble drying of the coated fabric which was not aged (Figure 3, comparison a2), showing that aging did have an impact on the compounds in the DWR. In Figure 3b, the effects of washing on 6:2 FTOH in the FC-6 coated PA fabric are shown for four different treatments. When the original coated material was washed plus tumble dried, the concentration of 6:2 FTOH increased from 92 μg/kg to 150 μg/kg (Figure 3, comparison b1). An increase of the 6:2 FTOH concentration was also observed when the concentration in the aged fabric (87 μg/kg) was compared with the concentration in the aged fabric, which was five times washed afterward without tumble drying (390 μg/kg) (Figure 3, comparison b2). The third comparison (Figure 3, comparison b3) shows an increase of the 6:2 FTOH concentration between the aged fabric (87 μg/kg), the aged fabric which was five times washed and tumble dried (430 μg/kg), and the aged fabric which was ten times washed and tumble dried (520 μg/kg). Those comparisons show that the increase of the concentration of 6:2 FTOH was caused by the washing process. This was confirmed by the results of the fourth comparison (Figure 3, comparison b4), in which the 6:2 FTOH concentration of the FC-6 coated PA fabric which was only tumble dried (6:2 FTOH 85 μg/kg) is compared with the concentration in the PA fabric when five washing plus tumble drying cycles were performed on the textile (150 μg/kg). The increase in 6:2 FTOH is most likely the result of transformation of FTOH precursors or side-chain cleavage due to, e.g., hydrolysis during washing.^54,57^

Tumble drying did not have an effect on the concentration of 6:2 FTOH in the PA fabric, as can be seen in the comparison of the 6:2 FTOH concentration in the original coated fabric with that in the fabric which was five times tumble dried (Figure 3, comparison c1). In the comparison of the concentration of 6:2 FTOH in the aged and washed fabric, which was not tumble dried with the fabric which was aged, washed, and tumbled dried (Figure 3, comparison c2), no difference was observed either.

An additional effect was observed for the combination of aging and washing. When five washing plus tumble drying cycles were performed on the original coated material, the concentration of 6:2 FTOH increased from 92 μg/kg to 150 μg/kg (Figure 3, comparison b1). When instead the five washing plus tumble drying cycles were performed on the aged fabric (87 μg/kg), the increase in the 6:2 FTOH concentration was almost three times higher (430 μg/kg) (Figure 3, comparison b3). As described above, aging by itself does not release 6:2 FTOH in the PA fabric, but this observation shows that washing does increase the extractable 6:2 FTOH concentration in the PA fabric, and a combination of aging and washing makes the extractable 6:2 FTOH fraction even larger. One of the mechanisms that could cause this higher increase in the extractable 6:2 FTOH concentration is the damaging of either the DWR coating or the fibers of the PA fabric as an effect of aging. Washing afterward causes the release of a larger NEOF fraction. Another mechanism would be the transformation of FTOH precursors by aging (e.g., oxidation) in combination with washing (hydrolyses), which has a large effect on the formation of FTOHs.

**Figure 4:**
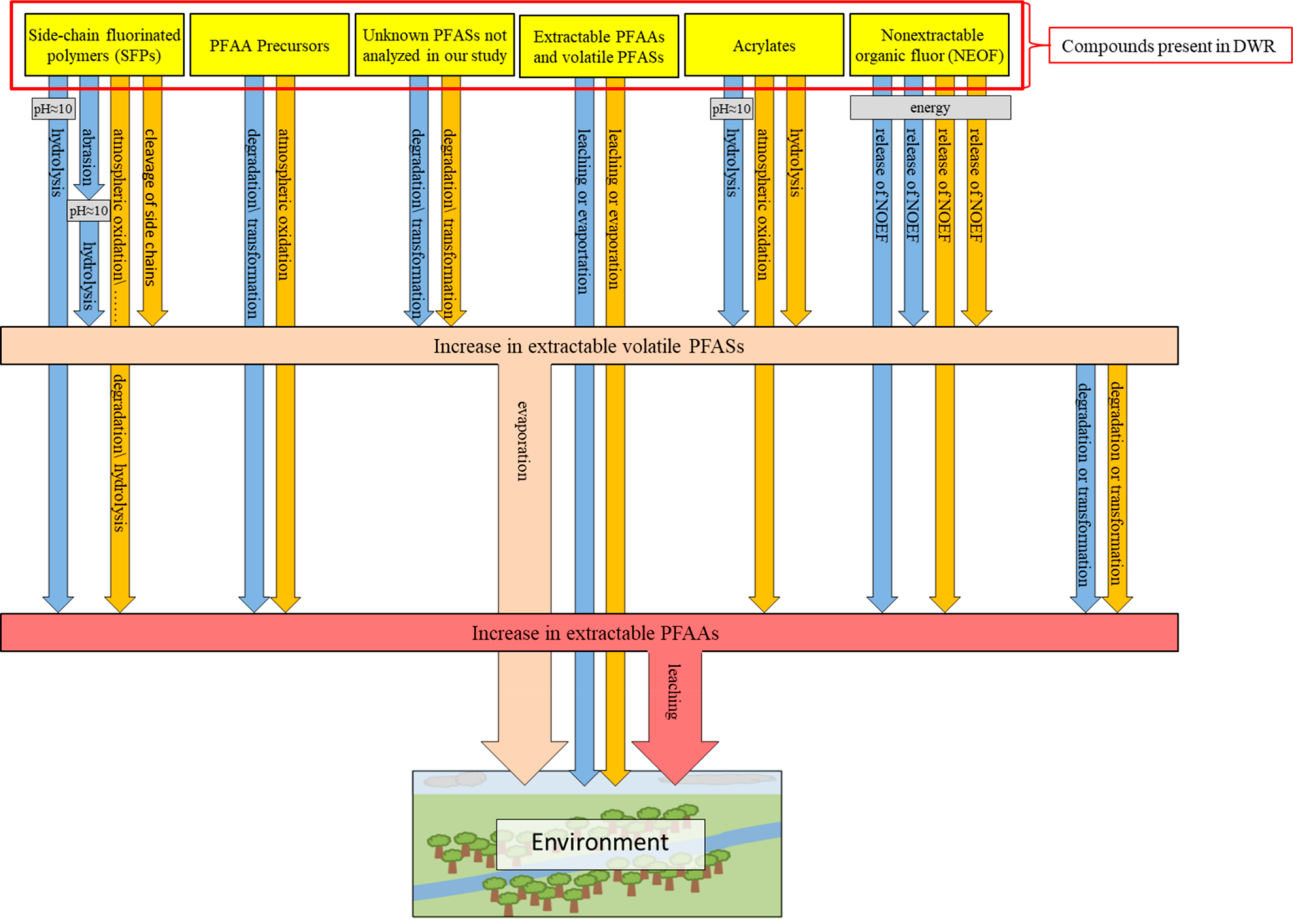
Potential mechanisms for the increase of extractable PFAS concentrations in and the emissions of PFASs from fabrics coated with the DWR based on SFPs as an effect of aging and washing of the fabrics: □ (blue), effect of washing; □ (orange), effect of weathering; and □ (yellow), compounds present in the DWR of fabrics.

An overview of all potential mechanisms for the increase of extractable PFAS concentrations in fabrics coated with the DWR based on SFPs and the emissions of PFASs from the fabrics as an effect of aging and washing of the fabrics is shown in Figure 4.

In conclusion, PFAS-based DWRs are not stable, and the stressors applied during the use phase contribute to the emission over time. The effects of aging, washing, and tumble drying on the concentrations of residual or unreacted PFASs in fabrics coated with the DWR based on SFPs are not just depending on the type of formulation and on the PFASs present in the textiles but also on the type of fabric. The PA fabrics and PES fabrics in our study which were coated with the same DWR emulsions contained different concentrations of PFASs. Volatile PFASs were found in higher concentrations in the PA fabrics than in the PES fabrics. Longer-chain PFAAs are not detected before and after aging on the FC-6 coated fabrics but are present on the FC-8 coated fabrics. Aging of the FC-6 coated fabrics, as well as of the FC-8 coated fabrics, resulted in an increase in PFAA concentrations. The effect of aging on the volatile PFASs was dependent on the type of fabric. An increase was observed on the PES fabrics, while no effect or a decrease was observed on the PA fabrics. Tumble drying on its own did not cause an observable effect, but washing either in combination with tumble drying or without tumble drying caused a decrease of the extractable PFAA concentrations. The PFAAs which are leached of short-chain PFAAs for the FC-6 fabrics and short- and longer-chain PFAAs for the FC-8 coated fabrics do end up in the sewage system. Via the sewage water treatment plant, the PFAAs finally end up in the surface water. The effect of washing on the volatile PFASs is dependent on the type of PFAS, the type of DWR, and the type of FC chemistry of the DWR coating. In general, volatile PFASs are emitted from the textiles, and the concentrations in the textiles decrease. However, washing can also cause the release of the unextractable fraction of volatile PFAS or the hydrolyses of FTOH precursors resulting in higher detected compounds in the fabric. This effect becomes stronger by a combination of aging and washing. The volatile PFASs which are detected on the fabrics after aging and washing can emit to the outdoor environment by evaporation when wearing the clothes or to the indoor environment when the clothes are hanging in the closet or on the coat rack.^60^ This increases the concentrations of PFASs in indoor environments and the exposure risk for consumers. Since the results in this study showed that aging and washing can increase the concentrations of PFAA congeners substantially in fabrics with SFP treatments, it can be concluded that a substance by substance regulation of PFAAs is not sufficient. The transformation of the PFAA precursor associated with production impurities and/or the degradation of SFPs results in a complex mixture of different PFAAs and other PFASs. Their occurrence is dependent on material combinations as well as the conditions of weathering and washing which makes the predictions of exact concentrations impossible. To close the mass balance on PFASs before and after aging, washing, and tumble drying, further research is needed with total fluorine analyses. Considering the results obtained in this study, the authors would strongly support the new proposal for a broad restriction under REACH covering all PFAS as a group.^61^

## Supporting Information Available

General compound, sample and technical information, homogeneity of PFASs in fabrics coated with DWR formulations, and PFAS concentrations (PDF)
