# Supplementary material for: Fate of Per- and Polyfluoroalkyl Substances from Durable Water-Repellent Clothing during Use
Source: Environ Sci Technol. 2022 Apr 11;56(9):5886–97. doi: 10.1021/acs.est.1c07876 (PMC9069696; doi:10.1021/acs.est.1c07876)
Supplement: Supplementary file 1 — es1c07876_si_001.pdf [file es1c07876_si_001.pdf]

Supporting Information

The fate of per- and polyfluoroalkyl substances (PFASs) from durable water repellent (DWR) clothing during use

Ike van der Veen*[a], Steffen schellenberger[b,c], Anne-Charlotte Hanning[d], Ann Stare[d], Jacob de Boer[a], Jana M. Weiss[b], Pim E.G. Leonards[a]

[a] *Department. Environment and Health (E&H), Vrije Universiteit, De Boelelaan 1085, 1081 HV, Amsterdam, The Netherlands*

[b] *Department Environmental Science (ACES), Stockholm University, Svante Arrheniusv. 8, SE-11418 Stockholm, Sweden*

[c] *RISE, Research Institutes of Sweden, Brinellvägen 68, 100 44 Stockholm, Sweden*

[d] *RISE IVF AB, Argongatan 30, SE-431 53, Mölndal, Sweden*

*Corresponding author: E-mail address: ike.vander.veen@vu.nl

Page number: 12

Figure number: 7

Table number: 6

Chapter 1 General compound, sample and technical information

The data in the Tables S1 and S2 show the general information of the compounds (table S1 PFAAs, table S2 volatile PFASs) assessed and analysed in this study. In table S3 information on the fabrics and the DWR formulation is given, and in table S4 the technical specifications and settings of the aging, washing and tumble drying methods are given.

Table S1 Full names, CAS numbers, acronyms, and chemical formula of PFAAs assessed and analysed in this study and their isotope-labeled ISs

Compounds	CAS No.	Abbreviation	Formula
Perfluorobutanoic acid	375-22-4	PFBA	C_3F_7COOH
Perfluoropentanoic acid	2706-90-3	PFPeA	C_4F_9COOH
Perfluorohexanoic acid	307-24-4	PFHxA	$C_5F_{11}COOH$
Perfluoroheptanoic acid	375-85-9	PFHpA	$C_6F_{13}COOH$
Perfluorooctanoic acid	335-67-1	PFOA	$C_7F_{15}COOH$
Perfluorononanoic acid	375-95-1	PFNA	$C_8F_{17}COOH$
Perfluorodecanoic acid	335-76-2	PFDA	$C_9F_{19}COOH$
Perfluoroundecanoic acid	2058-94-8	PFUnDA	$C_{10}F_{21}COOH$
Perfluorododecanoic acid	307-55-1	PFDoDA	$C_{11}F_{23}COOH$
Perfluorotridecanoic acid	72629-94-8	PFTrDA	$C_{12}F_{25}COOH$
Perfluorotetradecanoic acid	376-06-7	PFTeDA	$C_{13}F_{27}COOH$
Perfluorobutane sulfonate anion	45187-15-3	PFBS	$C_4F_9SO_3^-$
Perfluorohexane sulfonate anion	108427-53-8	PFHxS	$C_6F_{13}SO_3^-$
Perfluoroheptane sulfonate anion	375-92-8	PFHpS	$C_7F_{15}SO_3^-$
Perfluorooctane sulfonate anion	45298-90-6	PFOS	$C_8F_{17}SO_3^-$
Isotope-Labeled PFAAs			
Perfluoro-n-[1,2,3,4-$^{13}C_4$]butanoic acid	na	$^{13}C_4$-PFBA	
Perfluoro-n-[1,2,3,4,5-$^{13}C_5$]pentanoic acid	na	$^{13}C_5$-PFPeA	
Perfluoro-n-[1,2-$^{13}C_2$]hexanoic acid	na	$^{13}C_2$-PFHxA	
Perfluoro-n-[1,2,3,4-$^{13}C_4$]heptanoic acid	na	$^{13}C_4$-PFHpA	
Perfluoro-n-[1,2,3,4-$^{13}C_4$]octanoic acid	na	$^{13}C_4$-PFOA	
Perfluoro-n-[1,2,3,4,5-$^{13}C_5$]nonanoic acid	na	$^{13}C_5$-PFNA	
Perfluoro-n-[1,2-$^{13}C_2$]decanoic acid	na	$^{13}C_2$-PFDA	
Perfluoro-n-[1,2-$^{13}C_2$]undecanoic acid	na	$^{13}C_2$-PFUnDA	
Perfluoro-n-[1,2-$^{13}C_2$]dodecanoic acid	na	$^{13}C_2$-PFDoDA	
Perfluoro-1-hexane[$^{18}O_2$]sulfonate anion	na	$^{18}O_2$-PFHxS	
Perfluoro-1-[1,2,3,4-$^{13}C_4$]octane sulfonate anion	na	$^{13}C_4$-PFOS	

na = not available

Table S2 Full names, CAS numbers, acronyms, and chemical formula of volatile PFASs assesses and analysed in this study and their isotope-labeled ISs

**Compounds**	**CAS No.**	**Abbreviation**	**Formula**
4:2-Fluorotelomer alcohol	2043-47-2	4:2 FTOH	$C_4F_9CH_2CH_2OH$
6:2-Fluorotelomer alcohol	647-42-7	6:2 FTOH	$C_6F_{13}CH_2CH_2OH$
8:2-Fluorotelomer alcohol	678-39-7	8:2 FTOH	$C_8F_{17}CH_2CH_2OH$
10:2-Fluorotelomer alcohol	865-86-1	10:2 FTOH	$C_{10}F_{21}CH_2CH_2OH$
6:2 Fluorotelomer acrylate	17527-29-6	6:2 FTAC	$C_6F_{13}CH_2CH_2OC(O)CH{=}CH_2$
8:2 Fluorotelomer acrylate	27905-45-9	8:2 FTAC	$C_8F_{17}CH_2CH_2OC(O)CH{=}CH_2$
10:2 Fluorotelomer acrylate	17741-60-5	10:2 FTAC	$C_{10}F_{21}CH_2CH_2OC(O)CH{=}CH_2$
6:2 Fluorotelomer methacrylate	2144-53-8	6:2 FTMAC	$C_6F_{13}CH_2CH_2OC(O)C(CH_3){=}CH_2$
8:2 Fluorotelomer methacrylate	1996-88-9	8:2 FTMAC	$C_8F_{17}CH_2CH_2OC(O)C(CH_3){=}CH_2$
10:2 Fluorotelomer methacrylate	2144-54-9	10:2 FTMAC	$C_{10}F_{21}CH_2CH_2OC(O)C(CH_3){=}CH_2$
Isotope-Labeled volatile PFASs			
6:2 Fluorotelomer alcohol-D2	na	D_2-6:2 FTOH	
6:2 Fluorotelomer acrylate-D3	na	D_3-6:2 FTAC	
6:2 Fluorotelomer methacrylate-D5	na	D_5-6:2 FTMAC	

na = not available

Table S3 Fabrics and DWRs formulations (subtracted from Table S2 of Schellenberger et al. (2018)([1])

Fabric	Supplier	Chemistry	Specifiactions	
PA	FOV AB Borås, Sweden	Polyamide	115 +/- 5 g/m^2; black dyed, plain polyamide fabric with a square pattern ripstop design; ready for finishing; Threads per cm warp; weft= 60 +/- 1; 33 +/- 1	
PES	FOV AB Borås, Sweden	Polyester	120 +/- 5 g/m^2; black dyed, plain polyester fabric; ready for finishing; Threads per cm warp; weft= 60 +/- 2; 38 +/- 1	
DWR-Type	Supplier	Chemistry	Formulation	Curing conditions
FC-8	NDA*	C8 based SFP	1: 80 g/L C8-based polymer	1. Drying: 120°C; 3 min (wait for 10 min)<br>2. Curing: 175°C; 35 sec
FC-6	NDA	C6 based SFP	1: 80 g/L C6-based polymer<br>2: 10g/L Cross linker	1. Drying: 120°C; 3 min (wait for 10 min)<br>2. Curing: 175°C; 35 sec
FC-4	not stated	C4 based SFP	1: C4-based polymer<br>2: HC-based Extender	1. Drying: 120°C; 3 min<br>2. Curing: 160°C; 5 min

NDA = Non-disclosure agreement; SFP= side-chain fluorinated polymers

Table S4 Conditions of ATLAS weather-o-meter Cr 3000 for a weathering experiment (total duration 300 h).

Method:	A1 (ISO4892-2)
Exposure cycles:	102 min dry, 18 min water spray
Broadband (300-400 nm):	60 ± 2 W/m2
Narrowband (340 nm):	0.51 ± 0.02 W/m2·nm
Black standard temperature* (˚C):	65 ± 3 ˚C
Chamber temperature :	38 ± 3 ˚C
Humidity::	50 ± 10 %

* Reference temperature on a black metal plate in the ATLAS weather-o-meter Cr 300

Chapter 2 Homogeneity of PFASs in fabrics coated with DWR formulations

For a general assessment of the distribution of PFAS concentrations in fabrics coated with DWR emulsions by the method described by Schellenberger et al. 2018 [1], a homogeneity test is performed on the FC-4 coated PES fabrics (See Table S3).

For this, 20 samples were analysed out of one piece of FC-4 coated PES fabric (40x35 cm), and 10 samples out of another piece (40x35 cm) of FC-4 coated PES fabric. Out of all the relevant PFAA, only PFBA was present in quantifiable concentrations in this coated material. Results of the concentration and distribution of PFBA in the fabrics are given in table S5 and are shown on the corresponding spots of the fabric in figure S1. The mean concentration determined in the FC-4 coated PES fabrics was 23 µg/kg PFBA. The calculated relative standard deviation (RSD) of twenty analyses of samples originating from the first piece of fabric was 13%. The RSD of ten analyses of the second piece of fabric was 11%. The overall RSD of all 30 measurements was 14%. Although those RSDs are slightly higher than the RSDs (4-13%) of the repeatability determination of the analyses method as previously reported by Van der Veen et al. (2016) [2], the fabrics are homogeneous for PFBA on the 99% confidence level as determined with the soft CRM software [3].

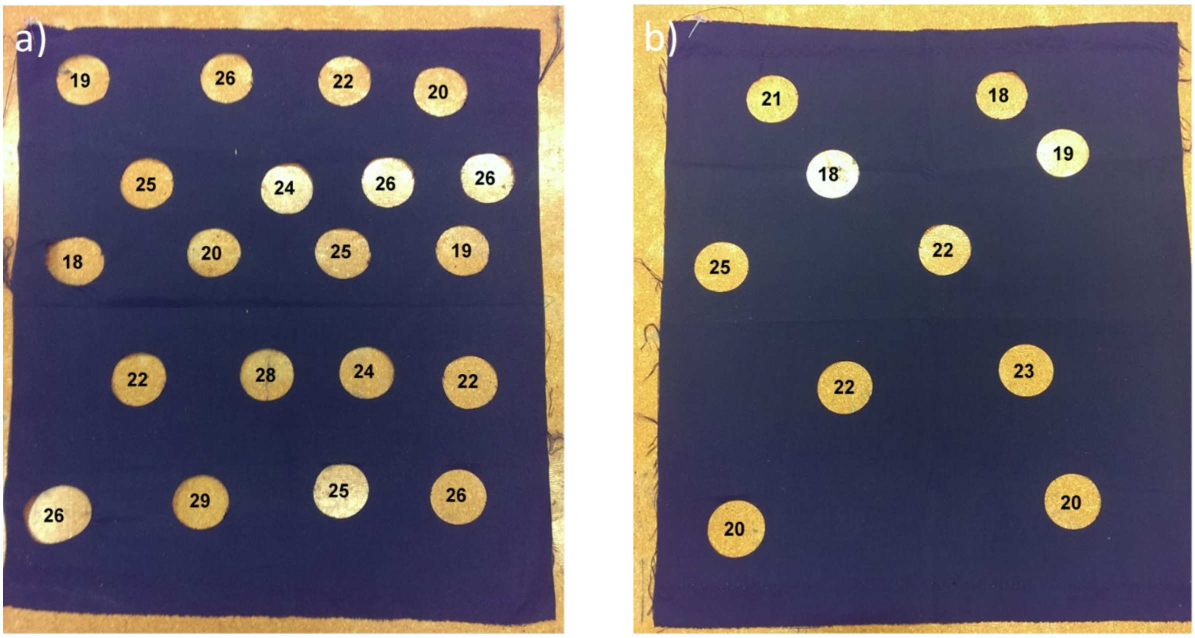

Figure S1 PFBA concentrations (µg/kg) determined in Fabric FC-4 PES represented on the spots the samples were taken from; a) Fabric 1; b) Fabric 2.

Table S5 *Results of the homogeneity test of PFASs in FC-4 coated PES fabrics.*

FC-4 PES	Sample no.	PFBA (µg/kg)
Fabric 1	1	19
	2	26
	3	22
	4	20
	5	25
	6	24
	7	26
	8	26
	9	18
	10	20
	11	25
	12	19
	13	22
	14	28
	15	24
	16	22
	17	26
	18	29
	19	25
	20	26
	Mean	24
	Stdev	3.2
	RSD	13%
Fabric 2	1	21
	2	18
	3	18
	4	19
	5	25
	6	22
	7	22
	8	23
	9	20
	10	20
	Mean	21
	Stdev	2.3
	RSD	11%
All samples	Mean	23
	Stdev	3.2
	RSD	14%

There was not enough material of the four types of repellent fabrics of interest in our study (FC-6 and FC-8 coated PA and PES fabrics) to perform all required experiments plus an extensive homogeneity test as has been performed on the FC-4 PES fabric. Instead the homogeneity of PFASs on those four types of repellent fabrics was assessed by cutting seven pieces (approximately 5*5 cm) out of each fabric for the analyses of PFAA (n= 5, no. 1- 5), and volatile PFASs (n=2, no. 6 and 7). The numbers of the samples are corresponding with the position on the fabric from where the samples were taken, as can be seen in the schematic overview of a piece of fabric in figure S2. PFAS concentrations determined in the samples are shown in figures S3 (FC-6 PA), S4 (FC-6 PES), S5 (FC-8 PA), and S6 (FC-8 PES), and are given in Table S6.

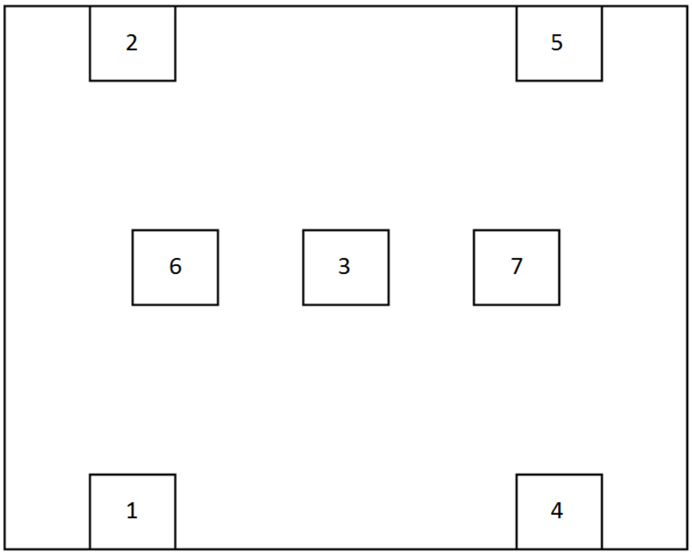

Figure S2 *Schematic overview of repellent fabric, representing the positions of which the samples are taken from for homogeneity testing of PFAAs (No. 1-5) and volatile PFASs (No. 6 and 7).*

Fabric FC-6 PA

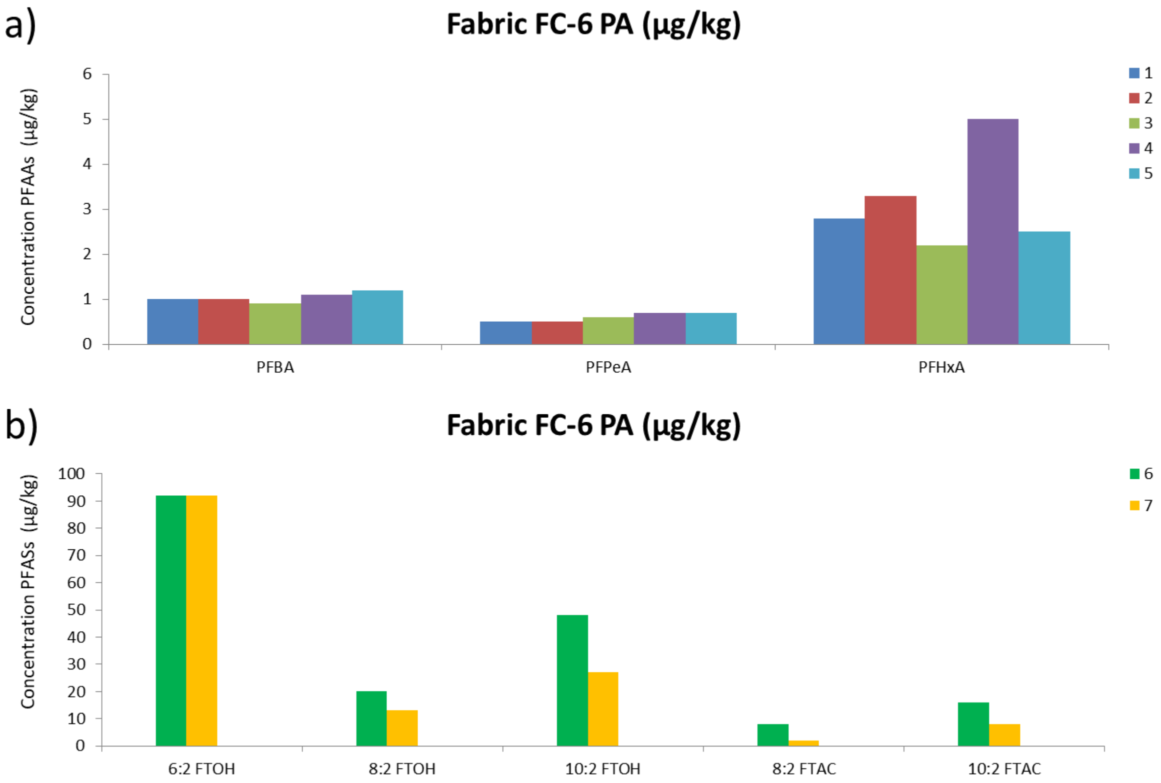

Figure S3 *PFAS concentrations (µg/kg) of Fabric FC-6 PA. The numbers 1-7 correspond with the positions of the samples on the fabric (see Figure S2); a) PFAAs (1-5); b)Volatile PFASs (6 and 7).*

In figure S3 the PFASs concentrations (a: PFAA, b: volatile PFASs) detected in samples of the FC-6 coated PA fabric are shown. In Fabric FC-6 PA three PFAAs (PFBA, PFPeA, and PFHxA) could be quantified, and five volatile PFASs (6:2 FTOH, 8:2 FTOH, 10:2 FTOH, 8:2 FTAC and 10:2 FTAC). For PFAAs, the RSDs over five samples were 11% for PFBA,

17% for PFPeA, and 35% for PFHxA with a difference of a factor 2.3 between the highest and the lowest concentration quantified for PFHxA. RSDs of the volatile PFASs in the two samples were 0% (6:2 FTOH) , 30% (8:2 FTOH), 40% (10:2 FTOH), 83% (8:2 FTAC) and 46% (10:2 FTAC). Except for 6:2 FTOH those RSDs are higher than those previously determined for the repeatabilities (0-28%, mean 7.7%) of the used analyses method [4]. The high inhomogeneity of the FC-6 coated PA fabric for those compounds has been taken into account with the evaluation of the results obtained within the aging and washing studies.

Fabric FC-6 PES

a)

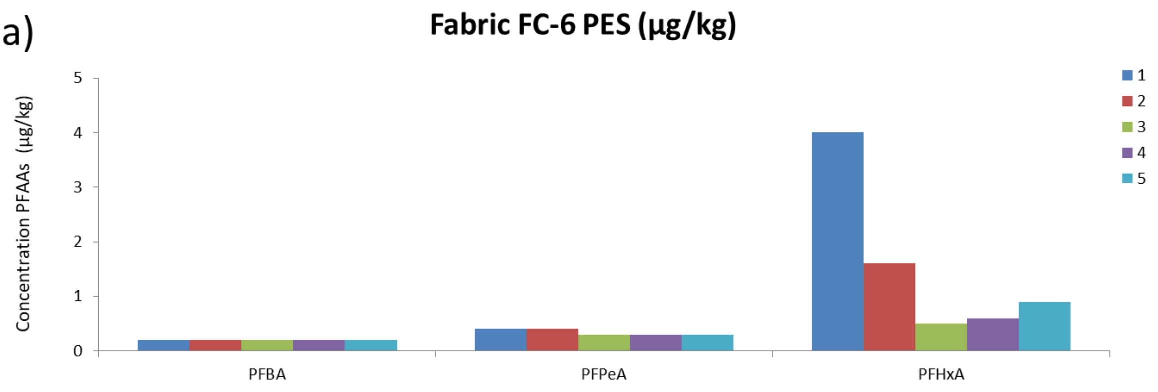

b)

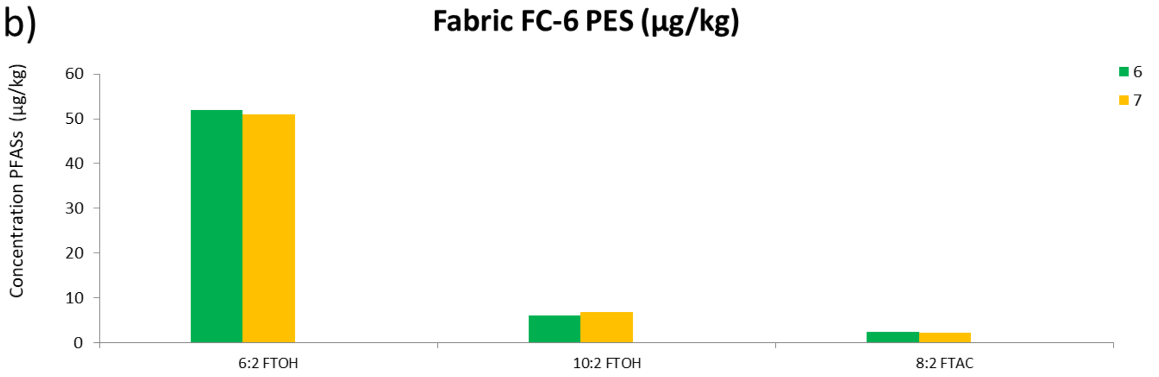

Figure S4 *PFAS concentrations (µg/kg) of Fabric FC-6 PES. The numbers 1-7 correspond with the positions of the samples on the fabric (see Figure S2); a) PFAAs (1-5); b)Volatile PFASs (6 and 7).*

In figure S4 the PFASs concentrations detected in seven pieces (approximately 5*5 cm) of Fabric FC-6 PES are shown. In Fabric FC-6 PES three PFAAs (PFBA, PFPeA, and PFHxA) could be quantified, and three volatile PFAS (6:2 FTOH, 10:2 FTOH and 8:2 FTAC). The RSDs over 5 samples were 0% for PFBA and 16% for PFPeA. For PFHxA the RSD was 95% with a difference of a factor 8 between the highest and the lowest concentration quantified. The RSD for the volatile PFASs are 1.4% (6:2 FTOH) , 8.8% (10:2 FTOH), and 6.1% (8:2 FTAC), which is lower than the RSD of the repeatability (0-28%, mean 7.7%) of the analyses method [4].

Fabric FC-8 PA

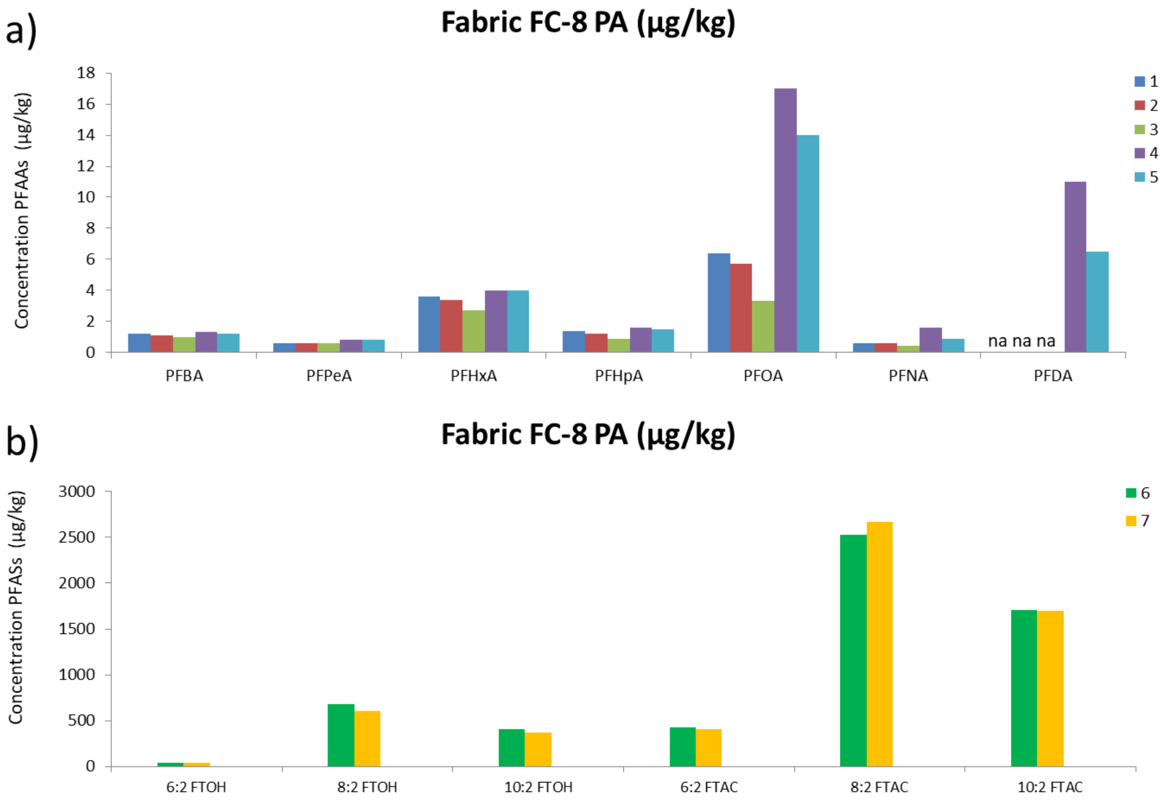

Figure S5 *PFAS concentrations (µg/kg) of Fabric FC-8 PA. The numbers 1-7 correspond with the positions of the samples on the fabric (see Figure S2 a) PFAAs (1-5); b)Volatile PFASs (6 and 7). (na = not available due to a low recovery of the labeled internal standard).*

In figure S5 the PFASs concentrations detected in samples of the FC-8 coated PA fabric are shown. Fabric FC-8 PA contained seven quantifiable PFAAs (PFBA, PFPeA, PFHxa, PFHpA, PFOA, PFNA, and PFDA), and six volatile PFASs (6:2 FTOH, 8:2 FTOH, 10:2 FTOH, 6:2 FTAC, 8:2 FTAC and 10:2 FTAC). PFDA could only be quantified in two of the samples (No 4, and 5), because the recovery of the labeled internal standard of PFDA ($^{13}C_2$-PFDA) in samples No 1,2 and 3 was too low to calculate an accurate concentration of PFDA. The relative standard deviations (RSDs) for PFBA, PFPeA, PFHxA, and PFHpA over five samples were all between 10 and 20%, which is higher than previously reported for the repeatability of the analyses method (0.1-8.7%) by Van der Veen et al. (2016) [2].

For PFOA a difference of a factor 5 has been quantified between the highest and the lowest concentration (RSD: 63%), and for PFNA a factor of 3.7 (RSD 57%). The high RSDs for those compounds in the analyses of Fabric FC-8 PA have been taken into account with the evaluation of the results obtained within the aging and washing studies.

For the volatile PFASs the RSDs over two samples were 0.0% (6:2 FTOH) - 7.7% (8:2 FTOH), which is lower than the repeatabilities determined for the method as reported in Van der Veen et al. (2020) [4].

Fabric FC-8 PES

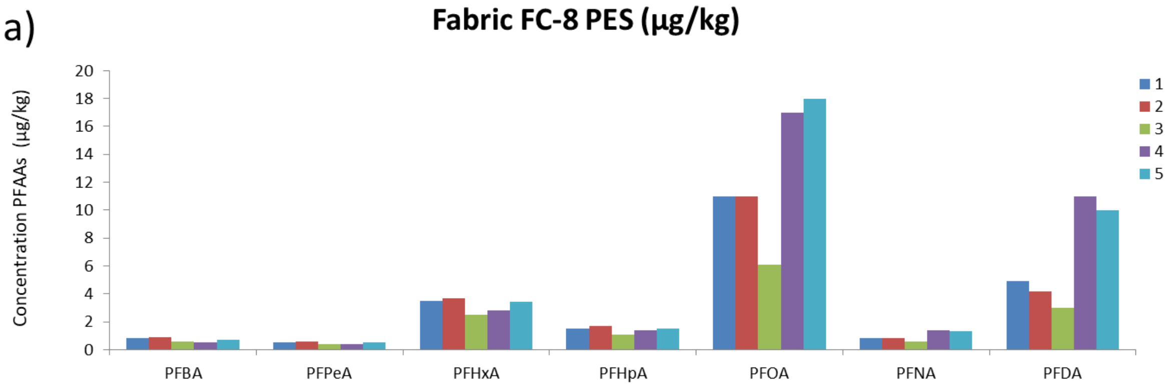

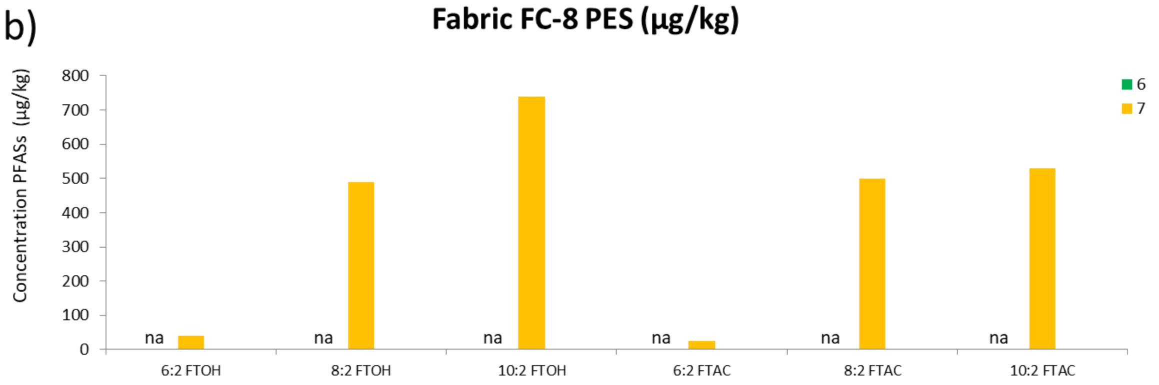

Figure S6 *PFAS concentrations (µg/kg) of Fabric FC-8 PES. The numbers 1-7 correspond with the positions of the samples on the fabric (see Figure S2); a) PFAAs (1-5); b)Volatile PFASs (6 and 7). (na= not available)*

In Figure S6 the PFASs concentrations of samples of Fabric FC-8 PES are shown. Fabric FC-8 PES contained seven quantifiable PFAAs (PFBA, PFPeA, PFHxa, PFHpA, PFOA, PFNA and PFDA), and six quantifiable volatile PFASs (6:2 FTOH, 8:2 FTOH, 10:2 FTOH, 6:2 FTAC, 8:2 FTAC and 10:2 FTAC). The RSDs of the PFAAs over five samples were 15-23% for PFBA, PFPeA, PFHxA, and PFHpA. For PFOA (RSD: 39%), PFNA (RSD: 36%), and PFDA (55%) the RSDs were higher with a difference of a factor 3.0, 2.3 and 3.7 respectively between the highest and the lowest concentration. Results showed that higher concentrations of those PFASs could be detected on the right side of the fabric. The RSDs determined here have been taken into account with the evaluation of the results obtained within the aging and washing studies. Since sample No. 6 was lost during analyses of the sample, the homogeneity of volatile PFASs on Fabric FC-8 PES could not be determined.

Conclusion

The homogeneity of PFASs in the four coated fabrics differ per compound, and per fabric. The RSDs of this limited homogeneity tests were taken into account with the evaluation of the results obtained within the aging and washing studies.

Chapter 3 PFAS Concentrations

PFASs concentrations are quantified in four DWR coated fabrics before and after weathering, washing and tumble drying experiments. In table S6 the PFAS concentrations in the original coated fabrics, and after the different experiments are given.

Table S6 PFAS concentrations quantified in DWR coated fabrics, before and after aging, washing and tumble drying experiments (µgkg)

		ionic PFAS																volatile PFAS										
	Sample description	Sample No	PFBA	PFPeA	PFHxA	PFHpA	PFOA	PFNA	PFDA	PFUnDA	PFDoDA	PFTrDA	PFTeDA	PFBS	PFHxS	PFHpS	Tot-PFOS	Sample No	4:2 FTOH	6:2 FTOH	8:2 FTOH	10:2 FTOH	6:2 FTAC	8:2 FTAC	10:2 FTAC	6:2 FTMAC	8:2 FTMAC	10:2 FTMAC
FC-6 PA	original	1	1.0	0.5	2.8	<0.2	<0.1	<0.1	<0.1	<0.1	<0.1	<0.1	<0.1	<0.1	<0.1	<0.1	<0.1	6	<2.8	92	20	48	<1.1	8.1	16	<4.5	<1.1	<1.1
		2	1.0	0.5	3.3	<0.2	<0.1	<0.1	<0.1	<0.1	<0.1	<0.1	<0.1	<0.1	<0.1	<0.1	<0.1	7	<2.7	92	13	27	<1.1	2.1	8.1	<4.4	<1.1	<1.1
		3	0.9	0.6	2.2	<0.2	<0.1	<0.1	<0.1	<0.1	<0.1	<0.1	<0.1	<0.1	<0.1	<0.1	<0.1											
		4	1.1	0.7	5.0	<0.2	<0.1	<0.1	<0.1	<0.1	<0.1	<0.1	<0.1	<0.1	<0.1	<0.1	<0.1											
		5	1.2	0.7	2.5	<0.2	<0.1	<0.1	<0.1	<0.1	<0.1	<0.1	<0.1	<0.1	<0.1	<0.1	<0.1											
		average	1.0	0.6	3.2	<0.2	<0.1	<0.1	<0.1	<0.1	<0.1	<0.1	<0.1	<0.1	<0.1	<0.1	<0.1	average	<2.8	92	17	38	<1.1	5.1	12	<4.5	<1.1	<1.1
	aged	8	3.7	8.8	34	7.6	<0.4	<0.3	<0.1	<0.4	<0.1	<0.1	<0.1	<0.1	<0.1	<0.1	<0.1	9	<5.7	87	<2.3	34	<2.2	<4.4	<2.2	<9.4	<2.2	<2.3
	aged + 5x washed and tumble dryed	10	<2.0	3.9	26	6.1	<0.2	<0.2	<0.1	<0.2	<0.1	<0.1	<0.1	<0.1	<0.1	<0.1	<0.1	11	<3.1	430	<1.2	18	<1.2	<2.3	<1.2	<5.0	<1.2	<1.2
	aged + 10x washed and tumble dryed	12	<1.9	2.5	20	4.2	<0.2	<0.2	<0.1	<0.2	<0.1	<0.1	<0.1	<0.1	<0.1	<0.1	<0.1	13	<3.3	520	<5.0	17	<1.3	<2.5	<1.3	<5.5	<1.3	<1.3
	aged + 5x washed, not tumble dryed	14	1.5	3.1	20	6.3	<0.3	<0.3	<0.3	<0.3	<0.1	<0.1	<0.1	<0.1	<1.7	<0.1	<0.2	15	<7.5	390	<1.7	<1.7	<1.1	<1.1	<1.1	<1.5	<1.5	<1.5
	not aged, 5x washed and tumble dryed	16	0.5	0.6	3.3	<0.5	<0.3	<0.3	<0.3	<0.3	<0.1	<0.1	<0.1	<0.1	<1.3	<0.1	<0.1	17	<5.0	150	<1.2	<1.1	<0.7	<0.7	<0.7	<1.0	<1.0	<1.0
	not aged, not washed, 5x tumble dryed	18	1.4	<0.2	17	<0.6	<0.3	<0.3	<0.3	<0.3	<0.1	<0.1	<0.1	<0.1	<1.5	<0.1	<0.1	19	<5.9	85	<1.4	<1.3	<0.9	<0.9	<0.9	<1.2	<1.2	<1.2
FC-6 PES	original	20	0.2	0.4	4.0	<0.1	<0.1	<0.1	<0.1	<0.1	<0.1	<0.1	<0.1	<0.1	<0.1	<0.1	<0.1	25	<2.8	52	<1.1	6.0	<1.1	2.4	<1.1	<4.7	<1.1	<1.1
		21	0.2	0.4	1.6	<0.1	<0.1	<0.1	<0.1	<0.1	<0.1	<0.1	<0.1	<0.1	<0.1	<0.1	<0.1	26	<2.9	51	<1.1	6.8	<1.1	<2.2	<1.1	<4.7	<1.1	<1.1
		22	0.2	0.3	0.5	<0.1	<0.1	<0.1	<0.1	<0.1	<0.1	<0.1	<0.1	<0.1	<0.1	<0.1	<0.1											
		23	0.2	0.3	0.6	<0.1	<0.1	<0.1	<0.1	<0.1	<0.1	<0.1	<0.1	<0.1	<0.1	<0.1	<0.1											
		24	0.2	0.3	0.9	<0.1	<0.1	<0.1	<0.1	<0.1	<0.1	<0.1	<0.1	<0.1	<0.1	<0.1	<0.1											
		average	0.2	0.34	1.5	<0.1	<0.1	<0.1	<0.1	<0.1	<0.1	<0.1	<0.1	<0.1	<0.1	<0.1	<0.1	average	<2.9	52	<1.1	6.4	<1.1	2.4	<1.1	<4.7	<1.1	<1.1
	aged	27	<3.1	<0.4	2.6	1.5	<0.4	<0.3	<0.1	<0.3	<0.1	<0.1	<0.1	<0.1	<0.1	<0.1	<0.1	28	<5.6	300	<2.2	15	<2.2	<4.2	<2.1	<9.1	<2.2	<2.2
	aged + 5x washed and tumble dryed	29	<2.0	2.6	10	2.3	<0.2	<0.2	<0.1	<0.2	<0.1	<0.1	<0.1	<0.1	<0.1	<0.1	<0.1	30	<3.4	430	<1.3	<6.3	<1.3	<2.6	<1.3	<5.6	<1.3	<1.4
	aged + 10x washed and tumble dryed	31	<1.7	2.2	7.9	1.9	<0.2	<0.2	<0.1	<0.2	<0.1	<0.1	<0.1	<0.1	<0.1	<0.1	<0.1	32	<2.8	460	<1.1	<3.9	<1.1	<2.1	<1.1	<4.6	<1.1	<1.1
FC-8 PA	original	33	1.2	0.6	3.6	1.4	6.4	0.6	na	na	na	na	na	<0.3	<0.2	<0.2	<0.6	38	<3.4	38	680	410	430	2540	1710	<5.6	<1.3	<1.4
		34	1.1	0.6	3.4	1.2	5.7	0.6	na	na	na	na	na	<0.3	<0.2	<0.2	<0.6	39	<3.5	38	610	370	410	2670	1700	<5.7	<1.4	<1.4
		35	1.0	0.6	2.7	0.9	3.3	0.4	na	na	na	na	na	<0.3	<0.2	<0.2	<0.6											
		36	1.3	0.8	4.0	1.6	17	1.6	11	na	na	na	na	<0.3	<0.2	<0.2	<0.6											
		37	1.2	0.8	4.0	1.5	14	0.9	6.5	na	na	na	na	<0.3	<0.2	<0.2	<0.6											
		average	1.2	0.7	3.5	1.3	9.3	0.8	8.8	na	na	na	na	<0.3	<0.2	<0.2	<0.6	average	<3.5	38	650	390	420	2610	1710	<5.7	<1.4	<1.4
	aged	40	3.8	4.6	12	11	29	12	11	4.0	5.8	<0.3	<0.1	<0.1	<0.1	<0.1	<0.1	41	<5.7	10	360	330	<2.2	6.5	<2.2	<9.3	<2.2	<2.3
	aged + 5x washed and tumble dryed	42	<2.0	1.6	5.7	5.1	15	4.3	5.1	1.1	2.1	0.2	0.3	<0.1	<0.1	<0.1	<0.1	43	<3.0	30	480	270	<3.0	5.1	<3.0	<3.0	<3.0	<3.0
	aged + 10x washed and tumble dryed	44	<2.0	<0.8	4.1	3.5	12	3.1	4.4	0.9	1.7	0.3	0.6	<0.1	<0.1	<0.1	<0.1	45	<3.5	13	480	260	<1.4	<2.7	<1.4	<5.8	<1.4	<1.4
FC-8 PES	original	46	0.8	0.5	3.5	1.5	11	0.8	4.9	<1.8	<7.0	<0.4	<1.8	<0.1	<0.1	<0.1	<0.4	51	na	na	na	na	na	na	na	na	na	na
		47	0.9	0.6	3.7	1.7	11	0.8	4.2	<1.7	<7.0	<0.4	<1.6	<0.1	<0.1	<0.1	<0.4	52	<2.9	41	490	740	26	500	530	<4.8	<1.1	<1.2
		48	0.6	0.4	2.5	1.1	6.1	0.6	3.0	<1.3	<7.0	<0.3	<1.1	<0.1	<0.1	<0.1	<0.4											
		49	0.5	0.4	2.8	1.4	17	1.4	11	<1.3	<7.0	<0.3	<1.3	<0.1	<0.1	<0.1	<0.4											
		50	0.7	0.5	3.4	1.5	18	1.3	10	<1.3	<7.0	<0.3	<1.5	<0.1	<0.1	<0.1	<0.4											
		average	0.7	0.5	3.2	1.4	13	1.0	6.6	<1.8	<7.0	<0.4	<1.8	<0.1	<0.1	<0.1	<0.4	average	<2.9	41	490	740	26	500	530	<4.8	<1.1	<1.2
	aged	53	<3.5	<0.5	<0.6	1.1	5.0	4.4	9.4	20	36	5.1	1.1	0.8	<0.1	<0.1	<0.1	54	<6.1	50	750	550	<2.4	8.3	<2.3	<10	<2.4	<2.4
	aged + 5x washed and tumble dryed	55	<1.9	<0.5	1.2	2.0	4.5	2.6	3.5	1.5	2.8	1.0	1.2	<0.1	<0.1	<0.1	<0.1	56	<3.4	69	510	350	<1.3	9.6	<1.3	<5.6	<1.3	<1.3
	aged + 10x washed and tumble dryed	57	<2.0	<0.4	0.8	1.4	3.6	1.6	3.3	0.9	2.5	0.5	0.8	<0.1	<0.1	<0.1	<0.1	58	<3.6	18	420	280	<1.4	<2.7	<1.4	<5.9	<1.4	<1.4

(na= not available due to low IS recovery)

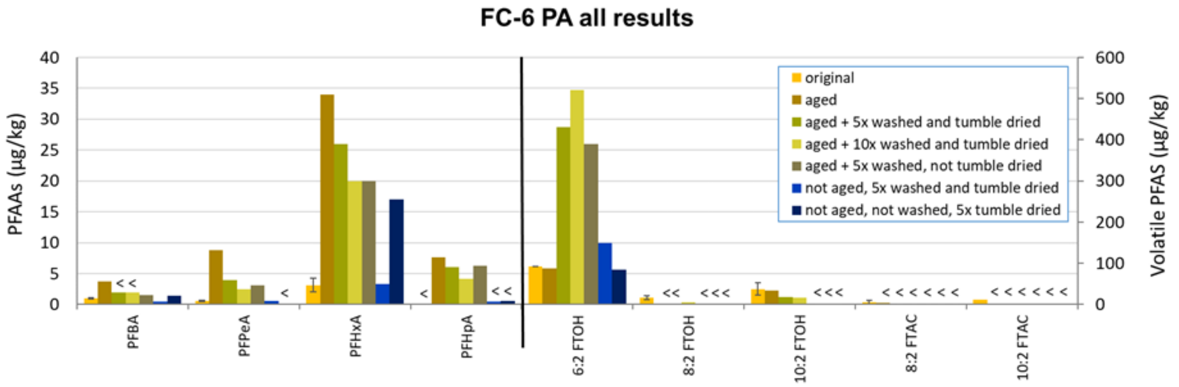

7

8 *Figure S7 PFASs concentration (µg/kg) in all analysed FC-6 coated PA samples. <: LOD.*

References

1. Schellenberger, S.; Gillgard, P.; Stare, A.; Hanning, A.; Levenstam, O.; Roos, S.; Cousins, I. T., Facing the rain after the phase out: Performance evaluation of alternative fluorinated and non-fluorinated durable water repellents for outdoor fabrics. *Chemosphere* **2018,** *193*, 675-684.
2. Van der Veen, I.; Weiss, J. M.; Hanning, A.; De Boer, J.; Leonards, P. E., Development and validation of a method for the quantification of extractable perfluoroalkyl acids (PFAAs) and perfluorooctane sulfonamide (FOSA) in textiles. *Talanta* **2016,** *147*, 8-15.
3. Bonas, G.; Zervou, M.; Papaeoannou, T.; Lees, M., ”SoftCRM”: a new software for the Certification of Reference Materials. *Accreditation and Quality Assurance* **2003,** *8*, 101-107.
4. Van der Veen, I.; Hanning, A.; Stare, A.; Leonards, P. E. G.; De Boer, J.; Weiss, J. M., The effect of weathering on per- and polyfluoroalkyl substances (PFASs) from durable water repellent (DWR) clothing. *Chemosphere* **2020,** *249*, 126100.
